# Functional Analysis of Promoters of Genes in Lipid Metabolism and Their Transcriptional Response to STAT3 under Leptin Signals

**DOI:** 10.3390/genes9070334

**Published:** 2018-07-03

**Authors:** Kun Wu, Xiao-Ying Tan, Yi-Huan Xu, Guang-Hui Chen, Mei-Qin Zhuo

**Affiliations:** 1 Laboratory of Molecular Nutrition, Fishery College, Huazhong Agricultural University, Wuhan 430070, China; pervcy@webmail.hzau.edu.cn (K.W.); xuyihuan@webmail.hzau.edu.cn (Y.-H.X.); cgh0626@webmail.hzau.edu.cn (G.-H.C.); zhuomeiqin@webmail.hzau.edu.cn (M.-Q.Z.); 2 Collaborative Innovation Center for Efficient and Health Production of Fisheries in Hunan Province, Changde 415000, China

**Keywords:** teleost, leptin, STAT3, promoter analysis, lipid metabolism

## Abstract

We characterized the promoters of target genes of the signal transducer and activator of transcription 3, STAT3 (carnitine palmitoyltransferase I, *CPT Iα1b*, acetyl-CoA carboxylase alpha, *ACCα*; fatty acid synthase, *FAS*; and peroxisome proliferator-activated receptor gamma, *PPARγ*) in a teleost *Pelteobagrus fulvidraco*. Binding sites of STAT3 were predicted on these promoters, indicating that STAT3 probably mediated their transcriptional activities. Leptin had no effect on the activity of *ACCα* and *PPARγ* promoters, but increased *CPT Iα1b* promoter activity and decreased *FAS* promoter activity. The −979/−997 STAT3 binding site of *CPT Iα1b* and the −794/−812 STAT3 binding site of *FAS* were functional binding loci responsible for leptin-induced transcriptional activation. The study provided direct evidence that STAT3 regulated the expression of *CPT Iα1b* and *FAS* at the transcription level, and determined the STAT3 response element on promoters of *CPT Iα1b* and *FAS* under leptin signal.

## 1. Introduction

Lipids are essential nutrients, and provide energy sources and essential fatty acids, which play important roles in numerous physiological and metabolic processes [1]. Excessive fat accumulation and disordered lipid metabolism have become serious problems in the sustainable and healthy development of aquaculture. In general, lipid homeostasis is characterized by the balance between lipogenesis and lipolysis. During lipogenesis and lipolysis, some crucial genes, transcriptional factors and enzymes are involved in these processes. Carnitine palmitoyltransferase I (CPT I) catalyzes the conversion of fatty acid-coenzyme A (fatty acid-CoA) into fatty acid-carnitines and is one of the limiting enzymes in fatty acid β-oxidation [2]. Both acetyl-CoA carboxylase alpha (ACCα) and fatty acid synthase (FAS) catalyze the committed steps in fatty acids’ biosynthesis [3,4]. Peroxisome proliferator-activated receptor gamma (PPARγ) is an important nuclear transcription factor that modulates the expression of many target genes relevant to lipid metabolism [5]. Many studies have been conducted to explore lipid metabolism [6]. However, information relevant to the modulatory mechanism of lipid metabolism at a transcriptional level is very scarce in fish. Thus, lipid metabolism-related factors and regulatory mechanisms in fish have attracted increasing attention.

Leptin is a member of the cytokines with the class-I alpha helix [7]. To date, many studies have been undertaken to explore the physiological roles of leptin, mainly focusing on the effects of leptin on food intake and energy homeostasis [7,8], but information about its molecular mechanism is still limited in teleosts [9,10]. Our previous studies have shown that leptin downregulated lipogenesis and upregulated lipolysis in yellow catfish by regulating the mRNA expression of the key lipid metabolism-related target genes, such as *CPT I*, *ACCα*, *FAS*, and *PPARγ* [11,12]. During these processes, signal transducers and activators of transcription protein 3 (STAT3) is one member of the Janus kinases (JAK)/STAT pathway and is considered the most important factor that transmits the leptin signal [12,13]. The activated STAT3 is capable of translocating to the nucleus to alter gene transcription [14]. Recently, using the specific JAK2/STAT3 inhibitor AG490, the effects of leptin on lipid metabolism were studied in grass carp [15], yellow catfish [11,12], and *Synechogobius hasta* [16]. These studies offered indirect evidence about the relationship between STAT3 and the regulatory role of leptin in genes involved in lipid metabolism. In fact, transcription initiation controls the expression of genes in eukaryotic organisms. Promoters contain cis-acting sequences and are bound by many transcriptional factors. Promoters control gene expression at the transcriptional level. Thus, as the first step in deciphering the mechanism of leptin-regulating target genes relevant to lipid metabolism, it is crucial to analyze the promoters’ structure and the function of downstream target genes. At present, studies on the promoters of lipid metabolism-related genes are scarce in fish. Recently, in our laboratory, Xu et al. [17] analyzed the structure and function of *CPT I* promoter in grass carp, but no studies have been reported for the promoter structure and function of *ACCα*, *FAS*, and *PPARγ* in teleosts.

Yellow catfish (*Pelteobagrus fulvidraco*) are omnivorous freshwater fish commonly farmed in some Asian countries for the delicious meat and high market value. The yellow catfish is considered a potential model fish for exploring the regulatory mechanism of lipid metabolism because it possesses high lipid contents in several tissues, such as the visceral tissues, liver, and muscles [18]. The present study was conducted to identify the promoter regions of *CPT Iα1b*, *ACCα*, *FAS*, and *PPARγ* in yellow catfish, and investigated the STAT3 binding sites of their promoter regions. Our study offers innovative insights into the mechanism of leptin regulating lipid metabolism and provides direct evidence on the interaction between STAT3 and downstream genes in fish.

## 2. Materials and Methods

### 2.1. Animals and Reagents

Juvenile yellow catfish were purchased from a commercial farm (Wuhan, China) and used for promoter cloning. HepG2 cell lines were from the Cell Resource Center of our college. Dulbecco’s Modified Eagle’s medium (DMEM), 0.25% trypsin-EDTA, and fetal bovine serum (FBS) were purchased from Gibco (ThermoFisher Scientific, Waltham, MA, USA). Recombinant human leptin (HPLC class) and other reagents were from Sigma–Aldrich (St. Louis, MA, USA). The Ethics Committee of Huazhong Agricultural University (HZAU) checked and approved our present experimental protocols on animals and cells (identification code: Fish-2016-0420, Date: 19 April 2016).

### 2.2. Promoter Cloning and Plasmid Construction

Based on our previous studies [5,19,20], we identified the 5′ cDNA sequences and the transcription start sites (TSS) of *ACCα* (GenBank number MH253822), *CPT1α1b* (GenBank number JQ074176), *FAS* (GenBank number MH253823) and *PPARγ* (GenBank number KF614118) of yellow catfish. The protocols of promoter cloning followed the method described in Xu et al. [17]. Briefly, genomic DNA was extracted from yellow catfish tail fins using a commercial kit (Omega, Norcross, GA, USA). In order to determine the position of the first intron of *ACCα*, *CPT1α1b*, *FAS*, and *PPARγ*, we designed various primers (shown in Table S1). The hiTAIL-PCR (high-efficiency thermal asymmetric interlaced-PCR) method [21] was used to clone the promoter sequences, and the specific primers with overlapping sequence are listed in Table S1. In order to produce the luciferase reporter constructs, we purified the PCR product and pGl3-Basic vectors (Promega, Fitchburg, WI, USA), and then digested them using endonucleases. Then we used ClonExpress II One Step Cloning Kit (Vazyme, Piscataway, NJ, USA) to ligate the products. Based on the distance from its TSS, we named the plasmids pGl3-2234/+51 of ACCα vector, pGl3-2155/+24 of CPT1α1b vector, pGl3-1960/+39 of FAS vector, and pGl3-2033/+63 of PPARγ vector, respectively. With the Erase-a-Base system (Promega) using templates of pGl3-2234/+51 of ACCα vector, we generated plasmids pGl3-402/+51, pGl3-793/+51, pGl3-1154/+51, pGl3-1518/+51, and pGl3-1914/+51 of ACCα vector. Similarly, using pGl3-2155/+24 of CPT1α1b vector as a template, we generated pGl3-387/+24, pGl3-726/+24, pGl3-1120/+24, pGl3-1480/+24, and pGl3-1709/+24; using pGl3-1960/+39 of FAS vector as a template, we generated the plasmids pGl3-326/+39, pGl3-746/+39, pGl3-1152/+39, pGl3-1380/+39, and pGl3-1525/+39; using template pGl3-2033/+63, we generated the plasmids pGl3-439/+63, pGl3-671/+63, pGl3-784/+63, pGl3-1241/+63, and pGl3-1575/+63, respectively. The primer sequences for plasmid construction are shown in Table S2.

### 2.3. Sequence Analysis

In order to analyze the promoter regions of *ACCα*, *CPT1α1b*, *FAS*, and *PPARγ* genes, we predicted the putative transcription factor binding sites (TFBS) using MatInspector online (http://www.genomatix.de/) and the JASPAR database (http://jaspar.genereg.net/). The reference binding site sequences are listed in Table S3. The Clustal-W multiple alignment algorithm was used to assess the sequence alignments.

### 2.4. Plasmid Transfections and Activities Assays of Luciferase

Plasmid transfections into HepG2 cells and activities assays of luciferase followed the methods described in our recent publication [17]. Briefly, HepG2 cells were cultured in DMEM medium + 10% FBS in an incubator with 5% CO_2_ at 37 °C. Plasmids were transiently transfected into HepG2 using Lipofectamine 2000 (Invitrogen, Carlsbad, CA, USA). The reporter plasmids were co-transfected with 35 ng pRL-TK as a control. After 4 h, DMEM (10% FBS) or DMEM (10% FBS) + 200 ng/mL leptin was used to replace the transfection medium. We chose the leptin concentration based on our publications [11,12,16]. Then, at the end of the 24-h incubation, we used the Dual-Luciferase Reporter Assay System to measure the relative luciferase activity, and the analytical protocols followed the manufacture’s manuals.

### 2.5. Site-Mutation Assays of STAT3 Binding Sites on the Promoter Regions of ACCα, CPT1α1b, FAS, and PPARγ

To identify the STAT3 binding sites on the promoter regions of ACCα, CPT1α1b, FAS, and PPARγ in yellow catfish, we used QuickChange II Site-Directed Mutagenesis Kit (Vazyme) to conduct site-directed mutagenesis. pGl3-ACCα-2234, pGl3-CPT1α1b-2151, pGl3-FAS-1960, and pGl3-PPARγ-2033 were used as the templates. The primers for mutagenesis are shown in Table S4. The constructs were named CMut-STAT3-1, CMut-STAT3-2, FMut-STAT3-1, FMut-STAT3-2, and FMut-STAT3-3, respectively. Then, we used Lipofectamine 2000 (Invitrogen) to co-transfect the pRL-TK and constructs into HepG2 cells. After 4-h transfection, we used DMEM (10% FBS) or DMEM (10% FBS) + 200 ng/mL leptin to replace the transfection medium. After a 24-h incubation, we harvested cells and the luciferase activity was determined based on the procedure mentioned above.

### 2.6. Electrophoretic Mobility-Shift Assay (EMSA) for Determining the Functional Binding Sites of STATs on the Promoter Regions

Proteins for electrophoretic mobility-shift sssay (EMSA) were extracted from HepG2 cells. EMSA assays were conducted to determine the functional binding sites of STAT3 on the promoter regions of *ACCα*, *CPT1α1b*, *FAS*, and *PPARγ*, based on the methods described by Xu et al. [17]. Cytoplasmic and nuclear extracts were obtained following the protocols of Read et al. [22]. Protein contents were determined following the bicinchoninic acid assay (BCA) method [23]. Each oligonucleotide duplex of STAT3 binding sites was incubated with 10 μg nuclear extracts according to LightShift Chemiluminescent EMSA Kit (Invitrogen), and each unlabeled probe was pre-incubated for 10 min prior to the addition of biotin-labeled probe. The reaction was allowed to proceed for 30 min after the addition of biotin-labeled probe at room temperature, and then they were detected by electrophoresis on 6% native polyacrylamide gels. Competition analyses were performed by using 100-fold excess of unlabeled oligonucleotide duplex with or without the mutation. These oligonucleotide sequences for EMSA were shown in Table S5.

### 2.7. Statistical Analysis

Results were presented as mean ± SEM (standard error of mean). Before analysis, we used the Kolmogornov–Smirnov test to determine the normality of distribution of all data. The Student’s *t*-test was used to compare the differences between the wild type (WT) and drug-treated group. Significance levels were set to *p* < 0.05. We used SPSS 19.0 software (SPSS, Chicago, IL, USA) to conduct the statistical analyses.

## 3. Results

### 3.1. Cloning and Sequence Analysis of the Promoter Regions of ACCα, CPT Iα1b, FAS, and PPARγ

In the present study, 2249 bp of *ACCα* promoter (GenBank accession No. MH253818), 2231 bp of *CPT1α1b* promoter (GenBank accession No. MH253819), 2022 bp of *FAS* promoter (GenBank accession No. MH253820), and 2171 bp of *PPARγ* promoter (GenBank accession No. MH253821) were cloned (Figure S1). The first nucleotide of 5′ cDNA of *ACCα*, *CPT Iα1b*, *FAS* and *PPARγ* was designated as +1. On the ACCα promoter region, we predicted several core promoter elements, such as a TATA-box (TBP) located from −24 bp to −40 bp and two CCAAT-boxes (nuclear transcription factor Y, NF-Y) located at −50 bp to −64 bp and −91 bp to −105 bp (Figure S2). We also predicted a cluster of binding sites of several transcription factors on the promoter region of *ACCα* gene, including HNF4α, STAT3, SREBP1, and PPARγ (Figure S2). On the core promoter of *CPT1α1b*, two GC-boxes (Sp1) were identified and there were two STAT3 binding sites located from −802 bp to −812 bp and from −979 bp to −997 bp (Figure S3). On the region of *FAS* promoter, we also discovered three STAT3 binding sites, which were located at the positions from –794 bp to −812 bp, −1571 bp to −1584 bp, and −1831 bp to −1845 bp, respectively. In addition, the binding sites of SREBP1 and PPARγ were also predicted (Figure S4). The binding sites of transcription factors such as STAT3, STAT5, CREB, and HNF4α were predicted on the region of *PPARγ* promoter (Figure S5).

### 3.2. 5′-Deletion Assay of the Promoter Regions of ACCα, CPT Iα1b, FAS, and PPARγ

**Figure 1:**
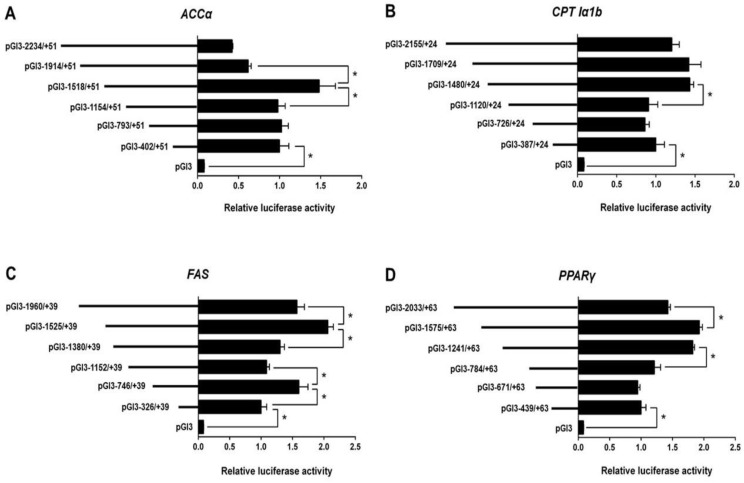
5′ unidirectional deletion assays of the promoter regions of *ACCα* (acetyl-CoA carboxylase alpha), *CPT Iα1b* (acetyl-CoA carboxylase alpha), *FAS* (fatty acid synthase,), and *PPARγ* (peroxisome proliferator-activated receptor gamma) of yellow catfish. (**A**) Assays for the *ACCα* promoter; (**B**) assays for the *CPT Iα1b* promoter; (**C**) assays for the *FAS* promoter; (**D**) assays for the *PPARγ* promoter. Values mean the ratio of activities of firefly to *Renilla luciferase*, normalized to the control plasmid. Results are shown as mean ± standard error of mean (SEM) (*n* = 3). Asterisk (*) means significant differences between two groups (*p* < 0.05).

According to the Erase-a-Base kit (Promega), we randomly generated plasmids of different size and selected six appropriate plasmids for each promoter to perform the deletion assay. The relative luciferase activity of the promoter of *ACCα* significantly increased when the sequence from −1518 bp to −1914 bp of *ACCα* promoter was absent. Subsequent absence to −1154 bp significantly reduced the relative luciferase activity (Figure 1A).

For the *CPT Iα1b* promoter, the sequence deletion from −2155 bp to −1480 bp showed no significant effects on luciferase activity, but the deletion from −1480 bp to −1120 bp reduced the luciferase activity (Figure 1B).

For the *FAS* promoter, the position deletion from −1960 bp to −1525 bp and −1152 bp to −746 bp upregulated the relative luciferase activity significantly (Figure 1C). In contrast, the position deletion from −1525 bp to −1380 bp and −746 bp to −326 bp downregulated the relative luciferase activity significantly.

For *PPARγ* promoter, the sequence deletion between −2033 bp and −1575 bp increased the relative luciferase activity significantly. In contrast, the deletion between −1241 and −784 bp reduced the relative luciferase activity significantly (Figure 1D).

**Figure 2:**
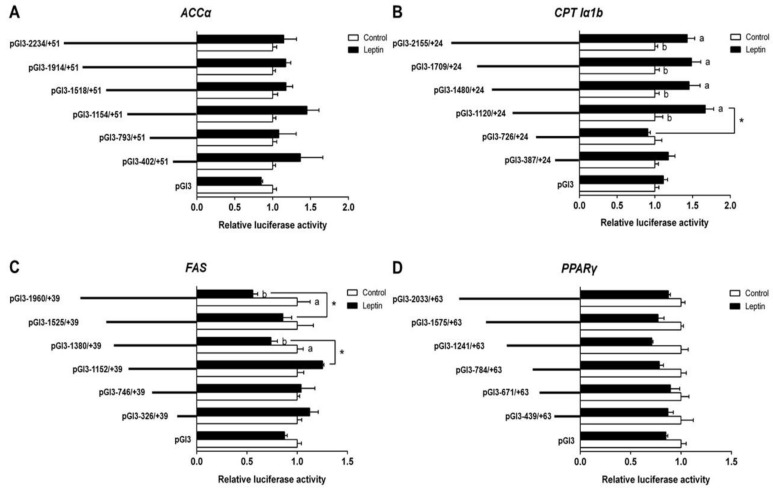
5′ unidirectional deletion assays for promoter regions of *ACCα*, *CPT Iα1b*, *FAS* and *PPARγ* after leptin treatment. (**A**) Assays for the *ACCα* promoter region; (**B**) assays for the *CPT Iα1b* promoter region; (**C**) assays for the *FAS* promoter region; (**D**) assays for the *PPARγ* promoter region. Values showed the ratio of activities of firefly to *Renilla luciferase*, normalized to the control. Results were presented as mean ± SEM (*n* = 3). Asterisk (*) indicates significant differences between different 5′ unidirectional deletion plasmids under the same treatment (*p* < 0.05). Different letters indicate significant differences between different treatments in the same plasmid (*p* < 0.05).

To investigate the response of promoters induced by leptin, we further used 200 ng/mL leptin to incubate HepG2 for 24 h and performed the 5′ deletion assay; the results are presented in Figure 2A. Compared with the control, leptin incubation did not significantly influence the relative luciferase activity of *ACCα*. No significant differences in relative luciferase activity were found between different deletion plasmid groups. In the leptin-treated group, the sequence deletion between −2155 bp and −1120 bp of *CPT Iα1b* promoter showed no significant influences on luciferase activity; however, further deletion to −726 bp downregulated the relative luciferase activity significantly. Meanwhile, the relative luciferase activity of the promoter region between −2155 bp and −1120 bp was significantly higher than that in the control (Figure 2B). Under leptin incubation, the region deletions between −1960 bp and −1525 bp, and −1380 to −1152 bp of *FAS* promoter significantly increased the relative luciferase activity. Leptin significantly decreased the luciferase activities of pGl3-1960/+39 and pGl3-1380/+39 (Figure 2C). Neither differences between different deletion plasmids nor differences between the control and the leptin-treated group were found in a deletion assay of *PPARγ* promoters (Figure 2D).

### 3.3. Site-Mutation Analysis of STAT3 Binding Sites on the Promoter Regions of CPT1α1b and FAS

**Figure 3:**
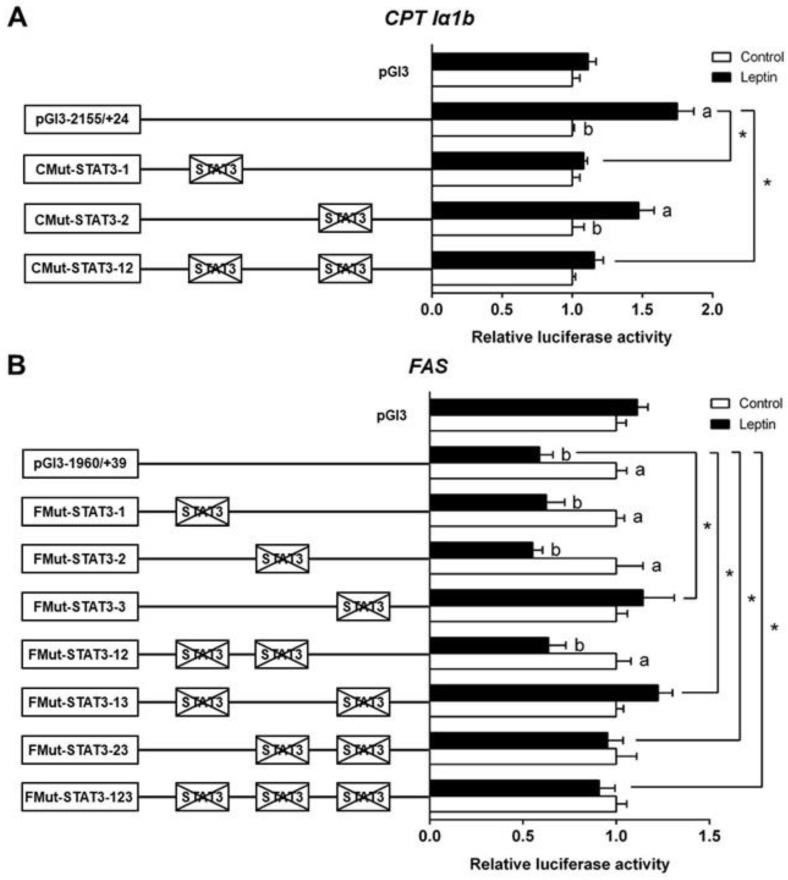
Assays of predicted STAT3 binding sites after site-directed mutagenesis. (**A**) Site mutagenesis of STAT3 on pGl3-CPTIα1b-2155 vector; (**B**) site mutagenesis of STAT3 on pGl3-FAS-1960 vector. Values mean the ratio of activities of firefly to *Renilla luciferase*, normalized to the control. Results were presented as mean ± SEM (*n* = 3). Asterisk (*) indicates significant differences between different 5′ unidirectional deletion plasmids under the same treatment (*p* < 0.05). Different letters indicates significant differences between different treatments in the same plasmid (*p* < 0.05).

Based on the results of 5′-deletion assay, we performed the site mutation in the regions of *CPT Iα1b* and *FAS* promoters that may possess STAT3 binding sites. The mutation of the −802/−812 STAT3 binding site (CMut-STAT3-2) did not change the leptin-induced elevation of luciferase activity, suggesting that this site played no role in the *CPT Iα1b* transcriptional response to leptin. The mutation of the −984/−994 STAT3 binding site (CMut-STAT3-1) downregulated the leptin-induced relative luciferase activity significantly. Similarly, the co-mutation of −984/−994 plus −802/−812 STAT3 binding sites (CMut-STAT3-12) also decreased the leptin-induced increase of luciferase activity, indicating that the −984/−994 STAT3 binding site positively mediated *CPT Iα1b* transcription (Figure 3A). For the *FAS* promoter, compared with the pGl3-1960/+39 vector (WT), the mutation of the −798/−808 STAT3 binding site (FMut-STAT3-3) significantly increased the relative luciferase activity, but the mutation of −1832/−1842 STAT3 binding site (FMut-STAT3-1) or −1572/−1582 STAT3 binding site (FMut-STAT3-2) did not significantly affect the relative luciferase activity in the leptin group. In contrast with the WT pGl3-1960/+39 FAS vector, the co-mutation of −798/−808 plus −1832/−1842 (FMut-STAT3-13), −798/−808 plus −1572/−1582 (FMut-STAT3-23) and −798/−808 plus −1832/−1842 plus −1572/−1582 (FMut-STAT3-123) STAT3 binding sites suppressed the leptin-induced decrease of luciferase activity (Figure 3B). The result reflected that the −1832/−1842 and −1572/−1582 binding sites could not regulate the transcription of *FAS* after leptin stimulation, and the −798/−808 STAT3 binding site negatively mediated the regulation of *FAS* transcription.

### 3.4. EMSA of Each STAT3 Binding Sequence

**Figure 4:**
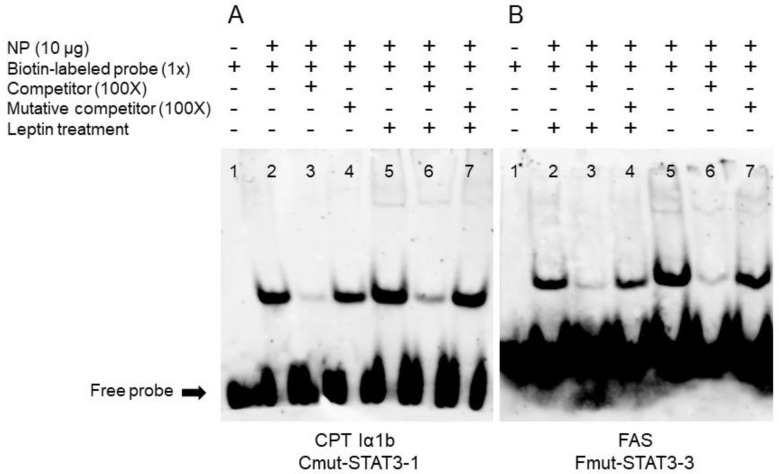
EMSA of predicted STAT3 binding sequence. (**A**) STAT3 binding sequences sited between −979 bp and −997 bp of *CPT Iα1b* promoter; (**B**) STAT3 binding sequences sited between −794 bp and −812 bp of *FAS* promoter. NP, nuclear protein.

Based on the results of the mutation assays above, we speculated that STAT3 could bind with the sequence from −979 bp to −997 bp of *CPT Iα1b* promoter and from −794 bp to −812 bp of *FAS* promoter. Therefore, we next used EMSA assay to measure their ability to physically interact with STAT3. When STAT3 binding sequence was used as the probe, the 100-fold unlabeled STAT3 binding sites (between −979 bp and −997 bp of CPT Iα1b promoter) competed for binding; in contrast, the 100-fold unlabeled Mut-STAT3 binding sites declined this competition significantly, indicating that this region could be bound by STAT3. Meanwhile, the increased brightness of bands under leptin treatment reflected that leptin promoted the binding process between STAT3 and this binding position on *CPT Iα1b* promoter (Figure 4A). Similarly, when the STAT3 binding sequence was used as the probe, the 100-fold unlabeled STAT3 binding site (between −794 bp and −812 bp of FAS promoter) competed for binding; in contrast, the 100-fold unlabeled Mut-STAT3 binding region downregulated this competition significantly, suggesting that this region could be bound by STAT3. In addition, compared with the corresponding bands in the non-leptin group, leptin reduced the brightness of bands, indicating that leptin mediated the transcriptional regulation of *FAS* by STAT3 (Figure 4B).

## 4. Discussion

Previous studies indicated that STAT3 played important roles in leptin-induced change of mRNA levels of lipid metabolism-related genes [11,12,16], but direct evidence of a link between STAT3 and lipid metabolism was not sought. The present study focused on several key genes (*ACCα, CPT Iα1b*, *FAS*, and *PPARγ*) that were regulated by leptin.

In our current study, one TATA-box and two CAAT-boxes were found in the core *ACCα* promoter region in yellow catfish. The TATA-box was commonly sited 25 bp upstream from the TSS and helps to identify the transcriptional initiation. In contrast, in humans, Mao et al. [24] reported that the *ACCα* core promoter regions possessed multiple Sp1 binding sites instead of the TATA-box or CAAT-box. The differences in core promoters between fish and humans reflect the complexity and diversity of transcriptional modulation among different species. In yellow catfish, the upstream of *CPT Iα1b* TSS contains neither a TATA box nor a CAAT box. Instead, we found Sp1 binding sites in the core promoter region. Studies suggested that TATA-less promoters usually possessed various Sp1 binding sites in their promoter regions [25]. Steffen et al. [26] also suggested that Sp1 could drive the basal expression of *CPT Iα* in the rat. Interestingly, *CPT Iα1b* core promoter of grass carp possesses a classical TATA-box, whereas in the promoter of *CPT Iα2a*, another isoform of *CPT Iα*, Sp1, and NF-Y replaces the TATA-box [17]. Thus, it is plausible that transcription initiation of various isoforms of CPT Iα gene presents diverse mechanisms. The core promoter region of yellow catfish *FAS* contains a classic TATA-box and CAAT-boxes. In contrast, in mammals, Amy et al. [27] reported that the transcription initiation of mammal FAS started with multiple Sp1. The present study indicated that yellow catfish had only one *PPARγ* promoter with the transcription initiation of classic TATA-box and CAAT-box. In the mouse, different splicing yielded two *PPARγ* promoters and both of the two isoforms contained a putative TATA-box [28]. In general, although the function of the same gene is similar between fish and mammals, the basic elements and pathways of transcription initiation of core promoters are not always conserved.

Our current study showed that the *ACCα* promoter of yellow catfish contained a SREBP1 binding site at −344/−353 bp and a *PPARγ* binding site at −366/−388 bp. Similarly, Mao et al. [24] reported that two SREBP binding sites were located about 230 bp upstream from the TSS in the human *ACCα* promoter regions. Moreover, the promoter regions at −402/+51 bp of *ACCα* positively modulated their promoter activities, suggesting that *SREBP1* and *PPARγ* were potential positive regulators for the activity of *ACCα* promoter. *SREBP1* and *PPARγ* are critical transcriptional factors for modulating lipogenesis [29], and they positively regulated *ACC*α mRNA expression [30,31]. On the yellow catfish *CPT Iα1b* promoter, we found a cluster of TFBS, such as HNF4α (−395/−419 bp) and *PPARα* (−348/−370 bp), in agreement with the structure of grass carp *CPT Iα* promoter [17]. Our study found that the sequence deletion from −387 bp to +24 bp significantly reduced the *CPT Iα1b* promoter activity. Considering that *PPARα* and *HNF4α* play important roles in regulating *CPT I* expression [32], *PPARα* and *HNF4α* may positively regulate *CPT Iα1b* promoter activity by combining the binding sites from −387 bp to +24 bp. The *SREBP1* and *PPAR* family are now well established as key transcription factors for the positive regulation of *FAS* expression [33,34,35]. In the present study, we found several binding sites of *SREBP1* and *PPARγ* on the FAS promoter. Furthermore, deletion of the regions for these binding sites significantly decreased the promoter activity. In addition, we found multiple STAT3 binding sites on the *FAS* promoter and the deletion analysis suggested that STAT3 was a potential negative regulator of *FAS* promoter activity; the *PPARγ* promoter of yellow catfish contained a CREB binding site at −1943/−1963 bp. Herzig et al. [36] pointed out that CREB was able to inhibit hepatic *PPARγ* expression. Our result showed that deletion of the region where CREB binding sites were located (−1575/−2033 bp) increased the *PPARγ* promoter activity, which further supports the regulatory role of CREB on *PPARγ* expression at the transcriptional level.

Recently, studies in our laboratory demonstrated the importance of STAT3 in regulating genes expression under leptin action [11,12,16]. However, information is scarce on the promoters of downstream target genes in fish, which precludes further speculation. Here, we analyzed the change of promoter activity using leptin incubation. Compared with the control, after deleting partial sequences of the *ACCα* and *PPARγ* promoters, the activity of promoters of *ACCα* and *PPARγ* remained relatively constant after leptin incubation. However, our previous studies indicated that leptin downregulated the expression of *ACCα* and *PPARγ* [11,12,16]. The apparent differences might be attributable to binding sites being located outside of the region we cloned. Compared with the control, leptin significantly increased the activity of *CPT Iα1b* promoter from −726 bp to −2155 bp, indicating that leptin promoted *CPT Iα1b* transcription, in agreement with previous studies in grass carp [15] and yellow catfish [11,12,16]. Studies suggested that leptin stimulated fatty acid oxidation [37,38]. CPT I is a rate-limiting enzyme for fatty acid β-oxidation. Taken together, initiating the *CPT I* transcription is the first and most important step by which leptin activates fatty acid oxidation. In addition, in the leptin-treated group, deletion of the sequence from −1120 bp to −726 bp of *CPT Iα1b* promoter significantly reduced the relative luciferase activity, indicating that the binding site that regulates *CPT Iα1b* transcription may fall in this region. For *FAS*, we found that leptin inhibited its promoter activity. *FAS* mediates the regulation of fatty acid biosynthesis [4]. Song et al. [11] indicated that leptin reduced its enzyme activity in vivo. Our study offered evidence that leptin downregulated *FAS* expression at the transcriptional level, which may be responsible for the reduction in its activity. Moreover, we identified two regions (−1960/−1525 bp and −1380/−1152 bp) related to regulation of the *FAS* promoter.

Having determined the key promoter regions that mediated the transcriptional regulation of *CPT Iα1b* and *FAS*, we next explored whether STAT3 exerts regulatory action directly through these response elements. According to the putative results, two STAT3 binding sites were located in the region from −1120 bp to −726 bp. However, in the *CPT Iα1b* promoter, site mutation on the −979/−997 STAT3 binding site, but not on the −802/−812 binding site, reduced the leptin-induced increase of promoter activities. Furthermore, an EMSA assay showed that the positions from −979 bp to −997 bp were a functional binding locus, and leptin promoted the binding of STAT3 to this site because of the stronger bands in the leptin-treated group. In the FAS promoter, we screened three possible STAT3 binding sites and found that leptin inhibited FAS gene expression via the STAT3 response element located from −794 bp to −812 bp on the FAS promoter. STAT3 is one member of the STAT family, which is implicated in programming gene expression [39]. Several studies pointed out that leptin administration caused the activation of STAT3 [11,12,15,16,40], and the activated STAT3 dimer subsequently entered the nucleus and regulated the transcription of target genes [41]. However, to the best of our knowledge, information is extremely scarce about the direct relationship between STAT3 and leptin-induced changes in lipid metabolism. For the first time, our study has indicated that STAT3 could directly bind with the promoter regions of CPT Iα1b and FAS, which potentially mediate the regulation of lipid metabolism by leptin.

## 5. Conclusion

In conclusion, we identified and characterized the promoter regions of *ACCα*, *CPT1α1b*, *FAS*, and *PPARγ* genes from yellow catfish. The promoters of *ACCα*, *CPT1α1b*, *FAS*, and *PPARγ* genes presented different structures on their core regions. 5′ deletion mutant analysis indicated the modulatory features of these promoters at the transcriptional level. Leptin treatment increased the activity of the *CPT1α1b* promoter and decreased the activity of the *FAS* promoter. Furthermore, leptin regulated the transcriptional activities of *CPT1α1b* and *FAS* through STAT3, and the functional binding locus of STAT3 on the promoter regions of *CPT Iα1b* and *FAS* genes was identified.

## Supplementary Materials

The following are available at http://www.mdpi.com/2073-4425/9/7/334/s1. Table S1: Primers used for ACCα, CPT1α1b, FAS, and PPARγ promoter cloning. Table S2: Primers used for 5′-deletion plasmids construction. Table S3: The reference binding site sequences. Table S4: Primers used for site mutation analysis. Table S5: Primers used for electrophoretic mobility-shift assay.
