# Supplementary material for: Functional Analysis of Promoters of Genes in Lipid Metabolism and Their Transcriptional Response to STAT3 under Leptin Signals
Source: Genes (Basel). 2018 Jul 3;9(7):334. doi: 10.3390/genes9070334 (PMC6071087; doi:10.3390/genes9070334)
Supplement: Supplementary file 1 [file genes-09-00334-s001.pdf]

Table S1. Primers used for *ACCα*, *CPT1α1b*, *FAS* and *PPARγ* promoter cloning

Gene	Forward primer (5'-3')	Reverse primer (5'-3')	Step
ACCα	CCATCCGAGTGAGGTTGTGG	AAGTGCTGTCCGAGTTGAGA	PCR
	ACGATGGACTCCAGAGCGGCCGCVVNVNNNCCAA	CGAGACGACTTGATGCAAGCTAATA	hiTAIL-PCR1
	ACGATGGACTCCAGAG	GAAATGAGTCGGCAAGCTATTCTGATGTGG	hiTAIL-PCR2
	ACGATGGACTCCAGAG	CACAACCTCACTCGGATGGATGTTCGTT	hiTAIL-PCR3
CPT1α1b	TGAACGCCAAATCGACATCCT	GTGGAGGCCTGACAGATAAAC	PCR
	ACGATGGACTCCAGAGCGGCCGCVVNVNNNCCAA	TTCGGTACTGATGCAGATTTAGGAT	hiTAIL-PCR1
	ACGATGGACTCCAGAG	CTAGGACGAAGAACGGTTCAGTTTAAGACAC	hiTAIL-PCR2
	ACGATGGACTCCAGAG	AAGTAATGGCTTCTCCGCCCTATGTAGTG	hiTAIL-PCR3
FAS	AAAGCAGTAAGAAGACCAAC	GCTATGACGATTTCCTCCATG	PCR
	ACGATGGACTCCAGAGCGGCCGCVVNVNNNCCAA	CTTAGGGTGTGAACTACTGACG	hiTAIL-PCR1
	ACGATGGACTCCAGAG	TTCTGCGTAGTCTAGACAAGC	hiTAIL-PCR2
	ACGATGGACTCCAGAG	CTGAGCTGCCTTGTAAGTCCT	hiTAIL-PCR3
PPARγ	TGGGATTTGGTCTAAGCACTC	TGTAGTTGTTCTCATGGGCTCT	PCR
	ACGATGGACTCCAGAGCGGCCGCVVNVNNNCCAA	CACGAAGCTGACGTAACAAGATGCTTTC	hiTAIL-PCR1
	ACGATGGACTCCAGAG	GACTTCCTGTTGCTTCACCCGCTGCTT	hiTAIL-PCR2
	ACGATGGACTCCAGAG	CATGTGGTTGTGCTGTGAGGTG	hiTAIL-PCR3

Table S2. Primers used for 5'-deletion plasmids construction

Gene	Primers	Forward primer (5'-3')	Reverse primer (5'-3')
ACCα	pGl3-402/+51	ctatcgataggtaccgagctcTCCATCCTCGTGTACCGTCA	cagtaccggaatgccaagcttCTCACTCGGATGGATGTTCG
	pGl3-793/+51	ctatcgataggtaccgagctcCTGTTCTTCCCAGATCTCACA	cagtaccggaatgccaagcttCTCACTCGGATGGATGTTCG
	pGl3-1154/+51	ctatcgataggtaccgagctcTCGGTATCTGAAGACTTGACTC	cagtaccggaatgccaagcttCTCACTCGGATGGATGTTCG
	pGl3-1518/+51	ctatcgataggtaccgagctcTTGCTCCGTTCTGCTAAATG	cagtaccggaatgccaagcttCTCACTCGGATGGATGTTCG
	pGl3-1914/+51	ctatcgataggtaccgagctcTGGATGGATGGATAAATGGAAGT	cagtaccggaatgccaagcttCTCACTCGGATGGATGTTCG
	pGl3-2234/+51	ctatcgataggtaccgagctcGTGTATACAGTAGATGTTGGCTT	cagtaccggaatgccaagcttCTCACTCGGATGGATGTTCG
CPT1α1b	pGl3-387/+24	ctatcgataggtaccgagctcTTAAATGGTTAGGTGTTAGGTGG	cagtaccggaatgccaagcttGCTTCTCCGCCCTATGTAGT
	pGl3-726/+24	ctatcgataggtaccgagctcCTGAAGAATCAAAGGAAAGGTG	cagtaccggaatgccaagcttGCTTCTCCGCCCTATGTAGT
	pGl3-1120/+24	ctatcgataggtaccgagctcGTTCGTGGGCATATTTGTTG	cagtaccggaatgccaagcttGCTTCTCCGCCCTATGTAGT
	pGl3-1480/+24	ctatcgataggtaccgagctcGCACAGACCTGATTGCTGA	cagtaccggaatgccaagcttGCTTCTCCGCCCTATGTAGT
	pGl3-1709/+24	ctatcgataggtaccgagctcCCCTAATTCTAACGAGCAAGCC	cagtaccggaatgccaagcttGCTTCTCCGCCCTATGTAGT
	pGl3-2155/+24	ctatcgataggtaccgagctcGGGATTAACTCTGTGGACTGG	cagtaccggaatgccaagcttGCTTCTCCGCCCTATGTAGT
FAS	pGl3-326/+39	ctatcgataggtaccgagctcCCTACAAGATGCAACACCTC	cagtaccggaatgccaagcttGGTCTTCTTACTGCTTTGGTT
	pGl3-746/+39	ctatcgataggtaccgagctcTCTGATTCGTTTACGGTCCTC	cagtaccggaatgccaagcttGGTCTTCTTACTGCTTTGGTT
	pGl3-1152/+39	ctatcgataggtaccgagctcATATAGCCGTTGCACTACACAG	cagtaccggaatgccaagcttGGTCTTCTTACTGCTTTGGTT
	pGl3-1380/+39	ctatcgataggtaccgagctcTGTAAATGACTTGTCAGCTCT	cagtaccggaatgccaagcttGGTCTTCTTACTGCTTTGGTT
	pGl3-1525/+39	ctatcgataggtaccgagctcGTACAAAATGTGCCATCCGAG	cagtaccggaatgccaagcttGGTCTTCTTACTGCTTTGGTT
	pGl3-1960/+39	ctatcgataggtaccgagctcTGTGCCAACATTTCACCCTG	cagtaccggaatgccaagcttGGTCTTCTTACTGCTTTGGTT
PPARγ	pGl3-439/+63	ctatcgataggtaccgagctcTGGTTATGGCACAAATGCAAC	cagtaccggaatgccaagcttTTGGTGATACTGAGCTTCGTAAT
	pGl3-671/+63	ctatcgataggtaccgagctcTTGGCTATAAATGCATCCTG	cagtaccggaatgccaagcttTTGGTGATACTGAGCTTCGTAAT
	pGl3-784/+63	ctatcgataggtaccgagctcCATGCCATGTTCCCTGGAC	cagtaccggaatgccaagcttTTGGTGATACTGAGCTTCGTAAT

pGl3-1241 /+63	ctatcgataggtaccgagctcTTGACTGGAAATCGGTTTATACT	cagtaccggaatgccaagcttTTGGTGATACTGAGCTTCGTAAT
pGl3-1575 /+63	ctatcgataggtaccgagctcTGGTAGGGAAGAGTACCTGA	cagtaccggaatgccaagcttTTGGTGATACTGAGCTTCGTAAT
pGl3-2033 /+63	ctatcgataggtaccgagctcTGCGGTGTCCAACATACTCA	cagtaccggaatgccaagcttTTGGTGATACTGAGCTTCGTAAT

Table S3. The reference binding site sequences

Name	Binding site sequences
CREB	TGAGCT
HNF4α	CAAAGT
MTF-1	TNTGCACACG
NF-κB	GGGANTTCC
NF-Y	CCAAT
PPARα	GGNAAAGGT
PPARγ	GGNAAAGG
SP1	CCCCNCCCC
SREBP1	TCACCCA
STAT3	TTCTNGGAA
STAT5	TTCCNNGAA
TATA-box	TATAAA

Table S4. Primers used for site-mutation analysis

Gene	Primers	Forward primer (5'-3')	Reverse primer (5'-3')
CPT1α1b	CMut-STAT3-1	CTAtcgaggtgtctTGTTTTTAATGTTTGCTGATTTTTTA	ACAagacacctcgaTAGCTGTTAAATAGCTAAATGGTGCA
	CMut-STAT3-2	AAAcaggatctgctAAGAAATAAAATAAATACACTGGCAACTAT	CTTagcagatcctgTTTAAATAATTATGTATGTAACTATTTACACCACT
FAS	FMut-STAT3-1	GAAacacattagagAGATTCCAGTACGATAACTTTACATGC	TCTctctaatgtgtTTCATATTTTTTAAATATGATGTTGATTAAAG
	FMut-STAT3-2	AGCcaccatacgaaGCAGTCAAGATTCGAAATTACAACA	TGCttcgtatggtgGCTTTGCCTTTTTACCAGGTTC
	FMut-STAT3-3	ACAtgcagctagctCCAAATTCCTTTTATGTGTCGACA	TGGagctagctgcaTGTGACCCTTAAAAGTACAGACATGG

Table S5. Primers used for electrophoretic mobility-shift assay

Primers		Forward primer (5'-3')	Reverse primer (5'-3')
CPT1α1b-STAT3-1	Biotin-probe	Biotin-CTAATTCACGTAAATGTTT	Biotin-AAACATTTACGTGAATTAG
	Mutative-competitor	CTATCGAGGTGTCTTGTTT	AAACAAGACACCTCGATAG
FAS-STAT3-3	Biotin-probe	Biotin-CACAAACACCTGGAACCAA	Biotin-TTGGTTCCAGGTGTTTGTG
	Mutative-competitor	CACAAGCAAGTCTATCGTA	TACGATAGACTTGCTTGTG

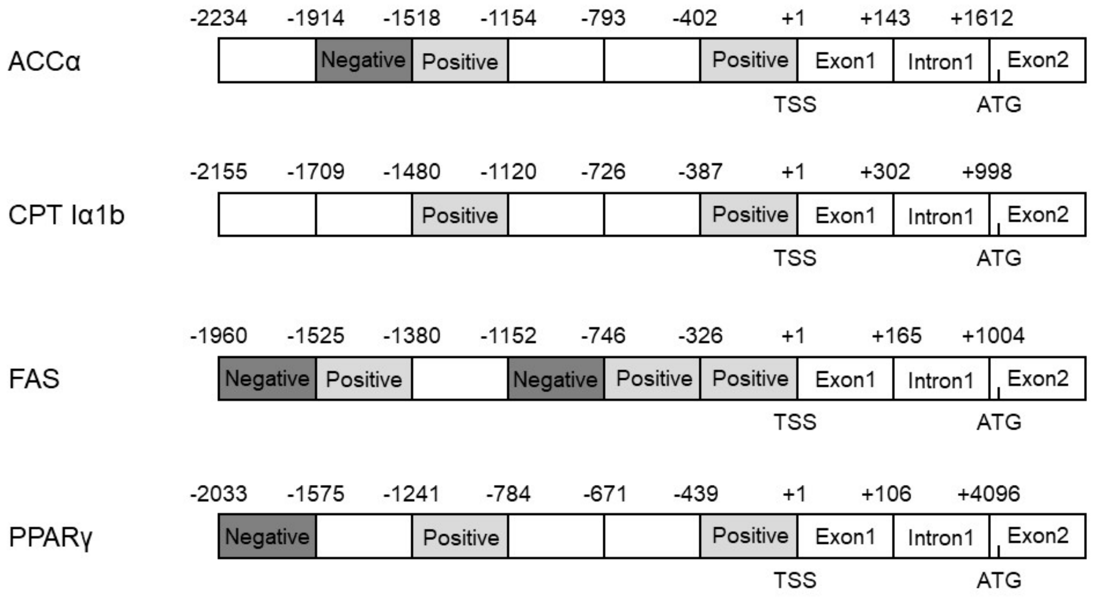

Figure S1 The schematic diagram of *ACCα*, *CPT Iα1b, FAS* and *PPARγ* gene structure. Positive: the region that positively regulated the promoter activity. Negative: the region that negatively regulated the promoter activity. TSS: transcription start site. ATG: translation initiation site.

```
CACAGCTCCA GTCTTGTGTA TACAGTAGAT GTTGGCTTTA AGCCCAACAC TTTTTTCCCC CTTTACTTAT CAGACAGTTT TCTGATAAAA AAAAAAAAAA -2150
AGAAATTGGA GAGCACAGTT ATCACAAGAA ACTATTACTA AACTGTGTGC TCTGGAAATG TACAAACAAA CATTCTGAAA TTCAGAGGAC ATTTAGTTTG -2050
TTGGACTTTC AGATTTCAGA AGTCAAAATG AGAACTAGCT CTTTATTCTT TTTTTTTTTT TTTTTTTAAA GATTCCATAA CTCTTATATT TCAGTAACTG -1950
     HNF4α
TAAAACAATT GCTGCACAAA AAATGGATGG ATGGATGGAT GGATGGATAA ATGGAAGTAA TAAGAAACAT GGTGAAATAA TTACAGATTT TTCCCATTTA -1850
TTGTGATGGT ATTTTTATAT GACACAAAAA CATATATGAA TCATTTACAT TACAGCTGAT ATAAACACTC GTTATAGCAA ACACTTGTTT ACTTCGTTTA -1750
      STAT3
TCAATTTTAC ATGAAAAGCA CTGACACTGT AGACTTCTTC CATAAATGTA AGACATTAGT CTTAGATTGC ATTGGGTCTT CAGTATACAT GTCCTTGTAA -1650
ACATCATACA CATCTTTAGT TGGTTACTAA CAATAACGTA TTAAAAGCAA ATCTGATCAA TCTGATAAGT GCTGCAATCA TTTTTTCAAA ACCCAGAATT -1550
GGTCAATGCA TAGGTTTTAA CTAGACTTAT TTTGCTCCGT TCTGCTAAAT GTTCACATAT AAATGATTTA TGATGAATGT TTTTATGATG ATGTTGACTG -1450
TTTTAAGTTC AGATTTCTTT TTGCCTGCTG AGAACATTTG AGGTGAATCA AGCTTTTGTT TATTTTTTCC TTCCATGAAT CCCTTCTAAG GTCTCATGTT -1350

CCACTGCCTG TTTTATCTGG CAGTAGGACA GGTGATGTCT GCATGATCAT TAAACTTTGA GCTGAACTTG AACCTAAAAA TCACGCTTGC ACAAGACTGA -1250
                                                                        PPAR/RXR
TATTTCATCG TTCTGAATCA ACCCGTTTAC ACAATAGTTA GATTGGATGC CAATGCCTCT GTGGTACGTC TGAGTGAACT CGGGTCGAAG GTGCTTCGGT -1150
ATCTGAAGAC TTGACTCTAT CTACAGTTAG CTGAAGTATT GAACTATTAT TTAAAAGACA TTTCATCCAA AGTGCCTTCC GGTTGAGCCT TGCAGATGAC -1050
GGTTAAAATC TACTGGGATT CGACCTCACA ACCTTCTGCT CGGTATTCCA TATTCAGTAT GATACTGACA CCGAAATGTT CCTTGATACC TAAGTAGATC -950
TTCTAGGTTG ATGGGATGTG ACTGTATCGG TGCATCAGTA CAACAGCACA CACATTAGCA TCATTATAAT GACTGCTGTG GAATGAAATA AAAATGTTCA -850
GTAATAGAGT TATGAATCAT GGCATTTTAC GTCATCCTCT TAAGAATGAT AAATATCTGT TCTTCCCAGA TCTCACAGAG ATGTCCATAC ATGTGTTCAC -750
TTTTTGGAAG GACAACTTTG GACATGTTTA ATACTTGGAG ACTGTTTGTT CTTTTGACCA ACTGAACCAC ATTATTATCC CAAATCGCTC GTCTTGTTAT -650
                                                      HNF4α
TGCAATGATA AGACATGCAA ACTTTTTGAA CTTATGTTAT TTGCATTACA TGTGATAATG TTATTTATTT TAGATCCCTA AAGCTATCTG AACCTTTGCA -550
CTCAATTATA TCTATAAAAA AAATAAGAAT AAATCATTAA ATCATTATCG GCAGTTCTTT TCCCCCTTGC TTATTATTGC TTTTTCTGTT TATTGCATTT -450
CTTATTGCAA ATTTTATTAT TTGTTATATA AATAAAATGG AAAAACATCC ATCCTCGTGT ACCGTCACCT TTAATTCATA AACAGATTTT AGTCGGATCA -350
                                                                         PPARγ
GGCGATACCG CGCACAAACA CGCGGACCAC CAGACCCTCT CTCTCATTGG TTACACCTCT CCTTATTTGC ATATCAAAAC CCGCCCCTAA CTGAAACACG -250
SREBP1
AAATTCTATT CGCTCATTCG TTCGGCGTGA GACTCTTAAA GTTCATAGTT CGACAATCTG CAGTAACCTC ACTCGACTCC CCGTTTTTAC ATCCCACTCG -150
CATTCCGACA TAACGCTTCC TTGTTTTGTG ATTGACTCAT ACAGCTGTCC AATCAGAGAC CAGAACTCGG GTGTACAATT TACATATGAC CAATCAGAGC -50
                                                       NF-Y                                  NF-Y
AAAACAGACC GGCAAATTCG AATACGGGTA GGATAAAAAA ACGCTTCCGG TGAGTAGCTA AGCTTATTAA CTCAGAAGCT AACGCTAGCT GAATATTAGC +51
                TATA-box                              TSS
```

Figure S2 Nucleotide sequence of yellow catfish *ACCα* promoter. Numbers are relative to the transcription start site (+1). The putative transcription factor binding sites are underlined. The highlighted sequences show putative transcription factor binding sites of STAT3.

```
CCTAACACTT TCTTTCCACT GACGTCTCTC CGGGGTTTAT TTTTGCCGAG AGGACTGCAA CAAATCTATG GACTGGAGTA GGGATTAACT CTGTGGACTG -2132
GAGTTGGGAT TAAATATGTG GACTGGAGAA ATTACTAAAT CTCTGGACTG GAGTACTGAC TAAATGTGTG GACTGGAGTA AAAAACTAAT TCATTCATTC -2032
ATTCGTTCAT TCATTCATTC ATTTTCTACC GCTTTTTCAA TTCAATTCAA TTTATTTGTA TATTTACTTT TAAAAATTCC CATTGTCTCA AAGCAGCTAT -1932
ACAGAAGTAT GGAAACAGAA GAGAGAGATA TAAATAAAAC AAACAAAAAA ACAAACAAAC AAACAAACAA ATAAAGAAAG AAAGAAAGAA AGAAAGAAAG -1832
AAGAAGAAGA AGAAGAAGAA GAAGAAGAAG AAGAACGGAT AAAAATAAAT AAATGAATAT ATACAATTAA AGTTTAAAAT GAATTTAAAA AAGATTACCT -1732
TAAAATTTAA AATACTATAT ATCTATCCCT AATTCTAACG AGCAAGCCGA AGGTGACTGC GGCAAGGAAA AACTCCCTGA GAAGATATGA GGAATAAACC -1632
TTGAGAGGAA CCAGACTCAG AAGGGAACCC ATCCTCATTT GGGTGACACT GAACAGGACA TAATGTAAAT GTAAAAAAAA AAAGTCCTTT CTACAACAGT -1532
TGATGCAACT GAGAGCTCCT GAAAAACTAA TGGGTCATCG TAAACTCTGA GTTCAGCACA GACCTGATTG CTGATCAAGA CCAGACCATA CAGTTGACTG -1432
ACACAACATT GATTCAAAAG AAGGCATGAT AATCTCTGGG CGGCTCCATC CACAACAATC CCATGAGACA CTTGCTGAAA ACAAGATTGT AGTAAAAAAC -1332
AAATTACTGC TGAAGTATTA AATACACCTA TGGATCAAAG TATTACTAAA TAAGTATATA TCTACACTCA CTGTCCACTT TATTAGAAAC ATTCATGCTT -1232
TTATTGAATC AGAGAAATAA CAGCAGCTCG ATTACTATGC ACCATAGTGA ACATTATATT TTAAATTTAA AATTGAATAT TAGGAATAGT GGACAGCAAT -1132
GGATTCCTTA CTATGGTTCG TGGGCATATT TGTTGCTTCA GCTCATAAAT CTGTTCTTTA TCGCAGTCGT TTTGTTTTGT TTTGTTTTGT TTAGTCCACA -1032
                 MTF-1                       STAT3
GGTTAAATCC TTGCACCATT TAGCTATTTA ACAGCTAATT CACGTAAATG TTTTTAATGT TTGCTGATTT TTTTAAACGT TAGAATAGTT CTGCACTAAA -932
TGGTTGTTAT TTTAAGACAA AAATAAATTT TGTACCCTAT ACAAAACTTT TTAATTTTAA GTCTTTAGTA TTAAATAAAT GAATAGTGGT GTAAATAGTT -832
                       STAT3
ACATACATAA TTATTTAAAC ATGTTGGAAT AAGAAATAAA ATAAATACAC TGGCAACTAT GTAAGCGTTA TTTTAATGTT TTTATTACAT ACTTACTTTT -732
CTTTCTATTC TGAAGAATCA AAGGAAAGGT GTATGAGACA GTAGTGAGAC CAGCTCTGCT GTATGGGTTA GAGACTGTAG CTGTGAGGAT CAGACATGAG -632
GCAGAGATGG CGGTAGCAGA TATGAGGATG TTGAGGTTCT CTTTAGGAGT GACAAGGATG GACAGGATTA GGAACGAGTG CATCAGAGGG ACGGCTCAGG -532
                                                                                MTF-1
TTGGCTGTTT TGTGGACAAG GTCAAAGAGG ATAGATTTAG ATGGTTTGGA CATGTACAAA TGAGGGAGAT TGTTATATTG GTAGAATGAT GTTGGGAATG -432
GAGGTGAGGT CAAGAGGAAG GACAAAGAGA AGATATATGG GCATGAAGTT AAATGGTTAG GTGTTAGGTG GAAACAGATT ATTTGCTGTG GCATTTTGGC -332
                   HNF4α                                              PPARα/RXR
TTTCTTTTTA TTCTCTTATT ACAAACTTGT CCCTCCTTTC ATCTGAGGGA TTCGATATTA TAAAAATAGC CACATGATTT ACATTGACAG GATTTTTTGT -232
                                 SP1
GTGAAACTTT GTGTGCTGCC ATTTTGGCCA GGACTCCCTG GTAGAAGAGA TTTTTTTTTA TCTCAATGGG ATTATTCCTG GTAAAATAAA AGATAAATAA -132

ATAAAAATAA ATAAATTTAT GCCTAAATTA ACAGCAGAAT AAATAAATAA ATCTCTTCCG CACCACTAGG GGGCGGTGTG CACATTAAGT AAGGTCGAGG -32
                                                                               SP1
TTGAATCCCA AAAATCTGTA ATGTAAACAA AGTGCACTAC ATAGGGCGGA GAAGCCATTA CTTTCTTTTA CTTATAGTGT GCCTATGTGC GGCGTGTGTA +69
                                  TSS
```

Figure S3 Nucleotide sequence of yellow catfish *CPT Iα1b* promoter. Numbers are relative to the transcription start site (+1). The putative transcription factor binding sites are underlined. The highlighted sequences show putative transcription factor binding sites of STAT3.

```
CAGTCCCTGC AATAAGCCAG GAGCTCAGTC AAATCTGCAT CTAGGCAGCT GTGTCACCAA AATGTGCCAA CATTTCACCC TGTTAACTTT TAGGTTTCAT -1913
                                                                                                 STAT3
AGATCTAAGT GGTTCAATTC AAGCACAGTT CTCAGTACCC TTCTGAACCT TTAATCAACA TCATATTTAA AAAATATGAA AATCCTAAAA GAGATTCCAG -1813
TACGATAACT TTACATGCAG GGTTACACAG CACACCATTT TGTGACCTTA TGTGTTGATG CCCCTGCATG GGCTTCCTCT GAATAATTTA CAGATACTGT -1713
TCCTGTGTCA GCCAGAGCAG AGGAAAATAG AAATACACCA CATCCCGGAT TACCAGTTCA AGTTCAGGGC TTGAAAGCAG TGATCCAGGA GAGAAACAAA -1613
                                              STAT3
AAAGCCAGAA CTACATCTGA ACCTGGTAAA AAGGCAAAGC CAGCTGGAAA GGCAGTCAAG ATTCGAAATT ACAACATTAA AGACTGAAAC TATGCAGGTA -1513
                          PPARα/RXR
CAAAATGTGC CATCCGAGCA AGATTTTGAA ACTATTTAAC AAACCCGGAT GTTGTGTTTT ATGAATCGTG AGTTCAAATA TGTGAAATAT TTGTTACCTT -1413
TACTGAACTC TAAACTCTGC GGGTTCCTTG TAAAATGTAC CTTGTAAATG ACTTGTCAGC TCTGTCATTG AAAATAAAAA ATGTTTTTGT AATAATAAAA -1313
PPARγ
TATTTTTATA CTCAATAACT CAATACTGTG GTTGTATTTT ACCTGCAAAG TATACACCTA CTTATGCCTA TAGTTTCTTA CACTTAGCAG TAAGGAAGTA -1213
TGTCGTGTGT CATCACCTGT CTAACCTGTC TCAAGTCAAG TCAAGGAGCT TTTATTGTCA TTTCAACCAT ATATAGCCGT TGCACTACAC AGTGAAATGA -1113
GACGTTTCTC CAGGACCATG GTGCTACACA AAACAAAGTG GCAATGGAGT GTTTTGAACA TAGTCGTGTA TGTAGCACAC TTTGGTTTCT CACTAGCTGC -1013
TTTATTATAC ACTCTATTTC AATCTACAAT CTGTGTTGCT TTCTGACCAC AGCACGGATG TTGGTTGCAT ATTTTTGTTT CAAAATGATC ACGAGATGTT -913
CAAACGCTTC TCAGACGAGC TGTTGGGCCT GATACAGATG TTCCAGCACC ACACATACAC ACAAACTCAA AGATGCCATG AGGTGGTGCC ATGTCTGTAC -813
                 STAT3
TTTTAAGGGT CACAAACACC TGGAACCAAA TTCCTTTTAT GTGTCGACAA ACTTGGCCAA TCAACCTGAT TCTGATTCTG ATTCGTTTAC GGTCCTCCAT -713
CCATGGACGC TGTGTGGTTA AACCGAGCAC TGAACGTGAA AGACTCTTGC GCTTTAACTA TAAACAGTAT AAAACATCTA TAGAGTGAAC CGAAATAACC -613
CCGTTAAACG ACACGCCCTC TTGATGACCT CACAGGCTAC GCTTGTCTTT AGCAGGACGC CTTAATCGGT TCGTCATCAC CTCACCCTAT ATCAGAAACA -513
                                                                                  SREBP1
CGCCCTTTCT TCGTCCTGAT TGGCCAAAAG TCAACGTCAA TCACGAAACT GTGTGATCTG CAAGCTGTCG CACGTGAAAC ATCCCAAAAT CACTGAAATA -413
                         PPARγ
AATGGCTTTC ACGCGACTAT TGTAAACTCA GTTTAACATT AATATATTTG TCCCTAGCTG ATTTAAAGCT TTTTGGAAAT AGCACTCGTA CATTTTCCTA -313
CAAGATGCAA CACCTCATTT TAAGACACAC ACACACACAA TGACGTCTAT ATCTAATTCT GACCCAGATT CATTAGGCAA TAAGGCAATG TACTTGTCTA -213
GGTTACTGTG ATAGCATGCA TCTGTGCATG ATGTTGCACA TAAACCTTCC ATTTCATGAC TGAACTCGAA TAACCTGTGT TTTTAGGCGG AATTAACGTT -113
AGGACATCAG CTCAGTGGTT GGATGTTGGA TCCCTGCCTC CTTTCTCATT GGACTAGAAA CCCAAGGCAA CTGTCAGCCC ATGTGGCGTG GCTATACAAC -13
               NF-Y                                   NF-Y
ATGGCACACA TTTAAATGAA ACAGACATCA GTGCAAAGGC AACCAAAGCA GTAAGAAGAC CAACACTCAG AAAAAAGGGG TTATTAATCC ACATTCACAG +78
         TATA-box       TSS
```

Figure S4 Nucleotide sequence of yellow catfish *FAS* promoter. Numbers are relative to the transcription start site (+1). The putative transcription factor binding sites are underlined. The highlighted sequences show putative transcription factor binding sites of STAT3.

```
GAGGAAGCTG AGGAAGCGCG GGAAACCTCA GGAGAGACAT CAACTGTTAA CTCATGCCTA TTAAAAGCAA TTGGAGGAAA GTTCATGTCC TCCGATATTG -2072
TGCAAGAGCA AATTCCCAAG GGGCTACACA ATAAAGATTG CGGTGTCCAA CATACTCATC AGAAAGGGTG TTTGCAGCAA ATCTGATTTG TCATCAAGGG -1972
                                                                 STAT5
TCTGGAGAGA ATGTTCAGGT AACCGATTGC TTAACATCCG TCCTCATGTA CTTGTGCATG TTTTAATGTT TTCTCCAGGG ATTTGGATTC AAAAGCATGA -1872
                CREB
GATCATAATT AGTCATGTGC TAGAACGTTA GAGGAGTTGC AATGTGCTTG CATAAATGTG TGTGTGTGCG TGTGTGTGTG TGCATGCGTG TGTGTGTGTG -1772
                                                                                     STAT3
TGTGCATGCG TGTGTGTGTG TGTGAGACGT TGCTTTGTGG TCTACAGCAG CTGATTAACT AGCTCAGGAT CATTTGCTTG AATTGCATTC TGTAAATGTC -1672
CAGTCAGCTG TTGTGGAAAG TTTGTACTTT TGTGGAAAGT TTGTACTTTT CACTTTTTTC TTTCCACTTT TCTAATGTTA ATGACAGCTT TTCTATTGGT -1572
AGGGAAGAGT ACCTGAATTG TATTAACTTA ATTTCACACT AAAACACAAA CTGAAAAAAA AAAATTACCT CACAAAAAAG GATTAAAATT ATTGGCAATA -1472
GGTGCATCCC AAATTTCAGT TTGCAATATT CTCTGCCATT TTGTAGTAAA AATTGTAGCA AGTTAACAGT GGAAATTCTA ACAAGTGGAA GTGCACTCGA -1372
                                                                             NF-κB
AGCACGCCGA TGACGCACTT ATTCAACCTA ATAAACAGCG AGTGGAATGA CACTTCAACC GTTGTAGCTT TCTTACACAA AAGGAAAGGG GCATTGAAAA -1272
AGTATAATTT TTTATGATGG CCACCGAACA TTGACTGGAA ATCGGTTTAT ACTTCCAGTG TAGTTTCACA TTTGAGACGA TACTCCCCTA AAATTCGTAC -1172
ACTAAACAGT AAAGTGCTTA GTGCAAGTGC TTAGTCATTT GGGACACAGC TGACGAGACT GGATTTTACC AAATTTTTAC ACATATATAT TCGTGTAACA -1072
AGCCCTCTTA TTTTTGGCAA CACCCACTGG ACAAATTATG CTAAGTTACC CAACATGGTG GAATGATCTA GAACAGGATA ACAGCAGATT TAGTGTTCCT -972
GGAATTGTAG CTTGTACCTA TTTAATTAAT TTAGATAAGT GGTTTACATG ACATATTATC CATATTCTCT AAAAGTCATG CAATCTTTTG TTTTTTTTTT -872
TGTTTCTCAG TCTGGGTGAA GTTGGGTGCA CCAGACTGCA GACTACTGCA GGCTTGTTAT TACAAAATGC TGTCTGTGTG AAAGACACAT GCCATGTTCC -772
CTGGACAAAA CCTAGTTTTA GCACAAAACC GTTTAGCAGA AAATGGTTCA TTTTAAATGG AGAAGGCTTT GTTATCTATT ATTATCTAGA AGCAGTGATG -672
TTGGCTATAA ATGCATCCTG CTTGAATAGA TTCTTTTTTT TTTAATCAGC GTATTTGTGG TATTTTGGTG TGTAACTTAA TATAATACTG TGTGTGTTCA -572
ATAACATGAT CTTTATTGCC AGTATTTGCA AATGCTTAGT GTGGCACGTG TGTAATATAT GGAATCATTT TTCCAGTAGG AATGAAATGT GTCTGTTAAC -472
                                                                                                HNF4α
TTGGTAAAAG TGCTTTTCGA ATCGTTTAAA CATGGTTATG GCACAAATGC AACATATTTC ACTATAAGAG TTTTGTAATA ACAAACACAG AACCAAATCA -372
AACACACACA CACACAAACA AACACACACA CACATACTGT ACAAAAAAAA TCTGATAGTA AAGCTACTAT CGGTCAACAT GACTGTCATG ATCATAGGAT -272
CTGAGGTCAG GGCCTCTCAG TAGTGACACT GAAAATGTCA CGGTGTAGAG AAAGCATCTT GTTACGTCAG CTTCGTGTTA TAAATTTATC CAGGGGAAGA -172
AGTGATCCAT CCAGCTTTCT GTAAAGAGAA GTGGAAAGCA GAAAAGCAGC GGGTGAAGCA ACAGGAAGTC ATCTGGCTAG CCGATCACCT CACAGCACAA -72
                                                                                       NF-Y
CCACATGATG GTTAGCTGTC TGCACTTTTG CTGAGGCCAT GCAGTGTAAA AATCAAGTTC CTGTTCAATT TGGTAGAACC ACTTGACCAG TTACCAAAGT +29
                                                  TATA-box                TSS
```

Figure S5 Nucleotide sequence of yellow catfish *PPARγ* promoter. Numbers are relative to the transcription start site (+1). The putative transcription factor binding sites are underlined. The highlighted sequences show putative transcription factor binding sites of STAT3.
